# A novel calcium-dependent mechanism of acquired resistance to IGF-1 receptor inhibition in prostate cancer cells

**DOI:** 10.18632/oncotarget.2346

**Published:** 2014-08-19

**Authors:** Cale D Fahrenholtz, Ann M Greene, Pedro J Beltran, Kerry L Burnstein

**Affiliations:** ^1^ Department of Molecular and Cellular Pharmacology, University of Miami, Miller School of Medicine, Miami, FL, USA.; ^2^ Oncology Research, Amgen Inc., Thousand Oaks, CA.

**Keywords:** insulin-like growth factor receptor 1, ganitumab, AMG 479, phospho-proteomics, VCaP, castration resistance

## Abstract

Inhibition of the mitogenic insulin-like growth factor receptor 1 (IGF-1R) signaling axis is a compelling treatment strategy for prostate cancer. Combining the IGF-1R inhibitor ganitumab (formerly AMG 479) with standard of care androgen-deprivation therapy greatly delays prostate cancer recurrence in xenograft models; however, a significant proportion of these tumors ultimately acquire resistance to ganitumab. Here we describe the development of a stable and reproducible ganitumab-resistant VCaP human prostate cancer cell derivative termed VCaP/GanR to investigate the mechanism of acquired resistance to IGF-1R inhibition. Unlike parental VCaP, VCaP/GanR did not undergo apoptosis following ganitumab treatment. VCaP/GanR did not express increased levels of IGF-1R, insulin receptor, or phospho-AKT compared to parental VCaP. VCaP/GanR exhibited increased levels of phospho-S6 indicative of increased mTOR activity. However, acquired resistance to ganitumab was not dependent on increased mTOR activity in VCaP/GanR. Phospho-proteomic arrays revealed alterations in several calcium-regulated signaling components in VCaP/GanR compared to VCaP. Reduction of intracellular calcium using cell-permeable calcium-specific chelators restored ganitumab sensitivity to VCaP/GanR through inhibition of cell-cycle progression. These data suggest a new mechanism of resistance to IGF-1R inhibition involving calcium-mediated proliferation effects. Such pathways should be considered in future clinical studies of IGF-1R inhibitors in prostate cancer.

## INTRODUCTION

Prostate cancer is the most commonly diagnosed cancer and the second leading cause of cancer-related deaths in U.S. men [1]. For decades, the standard care for advanced prostate cancer has been androgen-deprivation therapy. While this treatment regimen initially shows benefit and reduction in tumor volume, ultimately the tumors recur. Current treatments for recurrent disease are not sufficient and new therapeutic modalities are necessary to treat, prevent, or prolong time to progression.

The insulin-like growth factor receptor 1 (IGF-1R) is dysregulated in a variety of cancers including prostate cancer [2–5]. IGF-1R is a receptor tyrosine kinase (RTK) that plays an important role in mitogenesis, apoptosis, proliferation, motility and angiogenesis [6–9]. Thus, inhibition of the IGF-1 signaling axis is considered to be a potentially valuable approach for treatment of cancer [10].

Ganitumab (formerly AMG 479) is a fully human monoclonal antibody (IgG1) against IGF-1R [11]. Ganitumab binds the extracellular L2 domain of IGF-1R, which prevents the interaction of both native ligands, IGF-1 and IGF-2, to IGF-1R [12]. The IGF-1R is internalized and degraded after ganitumab binding [11, 13]. Ganitumab does not interact with the closely related insulin receptor (INSR) but has been shown to inhibit hybrid IGF-1R/INSR [11, 12]. Ganitumab inhibits several cell models of solid tumors both *in vitro* and *in vivo* [5, 11–14]. Recently ganitumab was examined in several phase II clinical trials alone and in combination with different chemotherapeutics for pancreatic and colorectal cancers with few dose limiting toxicities [15–20].

Recent clinical trials utilizing IGF-1R inhibition as prostate cancer therapy show favorable results. Treatment of naïve prostate cancer patients with figitumumab, an antibody inhibitor of IGF-1R, results in a marked decline in the biomarker prostate specific antigen (PSA) [21]. Combining another antibody inhibitor of IGF-1R, cixutumumab, with androgen-deprivation therapy shows significant changes in IGF and glucose homeostasis pathways [22]. These changes may result in conditions less favorable for tumor growth. These studies justify longer-term clinical trials and studies to assess the durability of IGF-1R inhibition as a treatment modality.

We previously showed that ganitumab decreases growth of well-established xenograft tumors representing both androgen-dependent and castration-resistant human prostate cancer [13]. IGF-1R inhibition is also effective in several other models of prostate cancer [23–26]. Combining androgen-deprivation therapy with ganitumab on established VCaP tumors (>300mm^3^) is most effective resulting in almost complete tumor regression that is maintained on average for 15 weeks. However, after long term ganitumab treatment, some tumors recur [13]. Therefore, it is imperative to investigate mechanisms of acquired resistance to ganitumab to improve ganitumab effectiveness in prostate cancer therapy.

In this study, we developed and characterized an *in vitro* model of acquired ganitumab resistance, which we termed VCaP/GanR using the VCaP prostate cancer cell line. VCaP are a human androgen-dependent prostate cancer cell line derived from a vertebral metastasis [27, 28] that harbors similar characteristics to human prostate cancer specimens including wild-type *PTEN* status (seen in approximately 50% of prostate cancers) [29] and expression of the *TMPRSS:ERG* fusion gene [30]. Using VCaP/GanR as a model, we evaluated the mechanism of acquired resistance to ganitumab. Unlike the parental VCaP, VCaP/GanR did not undergo apoptosis after ganitumab treatment; additionally, apoptosis was prevented in VCaP/GanR after serum starvation. While VCaP/GanR exhibited increased mTOR activity, attenuation of mTOR signaling was not sufficient to restore sensitivity to ganitumab. Lastly we found that acquired resistance to ganitumab in VCaP/GanR was dependent on intracellular calcium outlining a novel resistance mechanism that impacts cell proliferation through cell cycle alterations.

## RESULTS

### Development of a ganitumab resistant prostate cancer cell derivative

**Figure 1:**
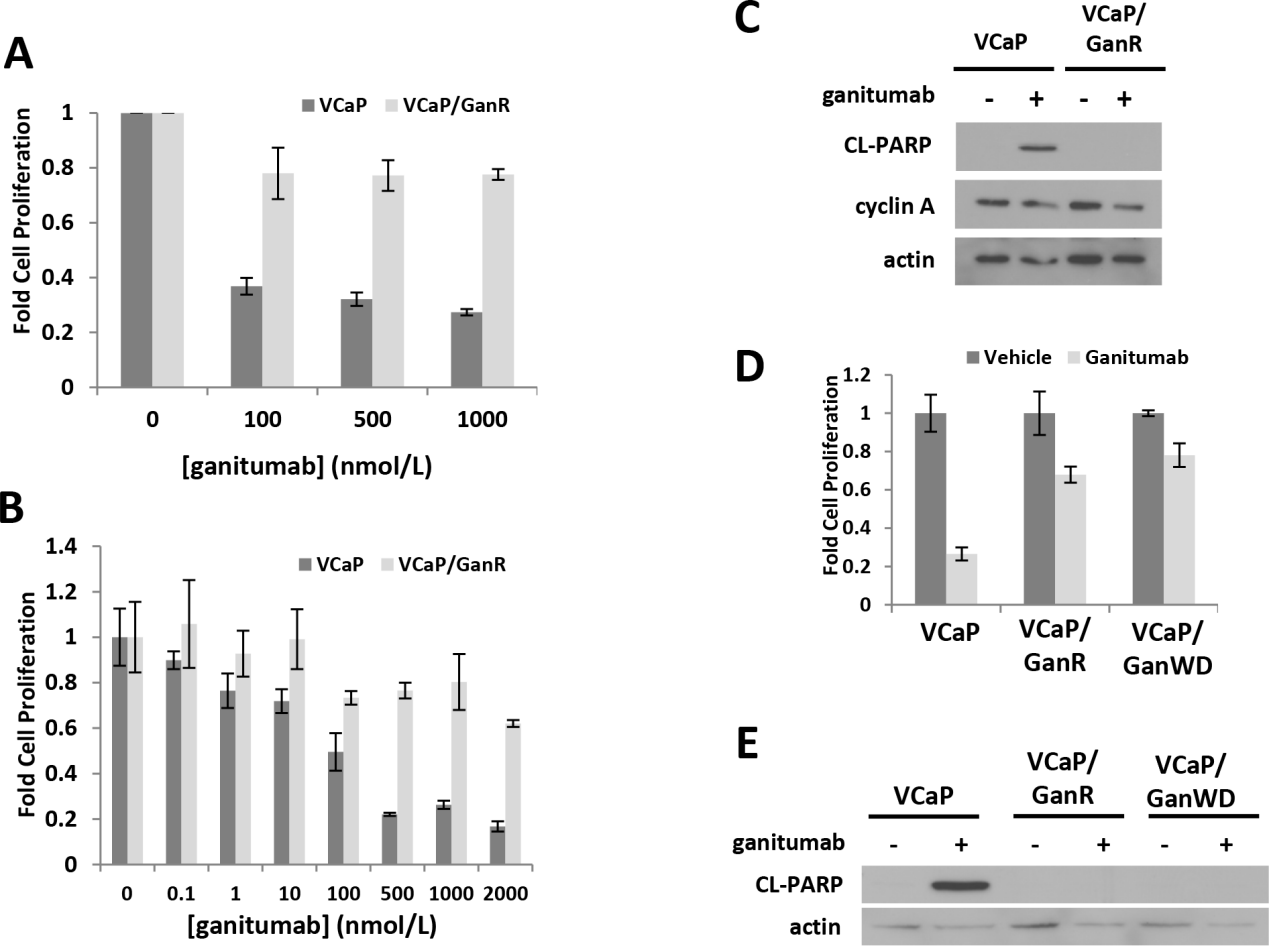
Characterization of a ganitumab resistant derivative of human prostate cancer VCaP termed VCaP/GanR VCaP and VCaP/GanR were treated with ganitumab **(A)** (0–1000 nmol/L) or **(B)** (0–2000 nmol/L) for six days in medium containing 2% FBS and proliferation relative to vehicle control is shown. **(C)** VCaP and VCaP/GanR were treated with ganitumab (500 nmol/L) or vehicle in medium containing 2% FBS for 72 hours and lysates were probed for cleaved PARP, cyclin A and actin. **(D)** VCaP, VCaP/GanR and VCaP/GanWD were treated with ganitumab (500 nmol/L) or vehicle for six days in medium containing 2% FBS and cell proliferation relative to vehicle treatment is shown. **(E)** VCaP, VCaP/GanR and VCaP/GanWD were treated with ganitumab (500 nmol/L) or vehicle in medium containing 2% FBS for 72 hours and probed for cleaved PARP and actin. Panel (A) represents three combined independent experiments performed in triplicate. Panels (B-E) are representative of at least 2 independent experiments. Data are shown ± SD.

To develop an *in vitro* model in which to examine mechanisms of resistance to ganitumab, VCaP were passaged in 500 nmol/L ganitumab for 12 weeks at which point significant cell proliferation was evident. These ganitumab resistant VCaP (termed VCaP/GanR) were routinely maintained in 500 nmol/L ganitumab. VCaP/GanR consisted of pooled clones that survived and proliferated following ganitumab treatment. Treatment of parental, passage-matched VCaP with ganitumab significantly decreased cell proliferation compared to VCaP/GanR (Figure 1a). Even at higher concentrations of ganitumab (2000 nmol/L), VCaP/GanR were not substantially growth inhibited (Figure 1b).

To determine whether the anti-proliferative effects of ganitumab were due to decreased proliferation or increased cell death, ganitumab was administered to parental VCaP and VCaP/GanR and levels of cleaved PARP, a marker of apoptosis, and cyclin A, a marker of cell cycle G1 to S phase progression, were assessed. Ganitumab increased cleaved PARP in parental VCaP, but little cleaved PARP was evident in VCaP/GanR and there was no change with ganitumab treatment (Figure 1c). In contrast, ganitumab treatment modestly reduced cyclin A levels in both cell lines. An additional ganitumab resistant cell model (termed VCaP/GanR-2) was derived in the same manner as VCaP/GanR and exhibited a similar pattern of resistance to ganitumab (Supplementary Figure 1a, b).

To determine whether the acquired resistance to ganitumab was a stable change, ganitumab was withdrawn from VCaP/GanR for 8 weeks (termed VCaP/GanWD). VCaP/GanWD were then rechallenged with ganitumab and found to maintain equivalent resistance to ganitumab as VCaP/GanR assessed by cell proliferation assays (Figure 1d). Additionally, ganitumab did not increase levels of cleaved PARP in VCaP/GanWD suggesting that acquired resistance to ganitumab was a stable phenotype (Figure 1e).

### Evaluation of androgen dependent status of VCaP/GanR

**Figure 2:**
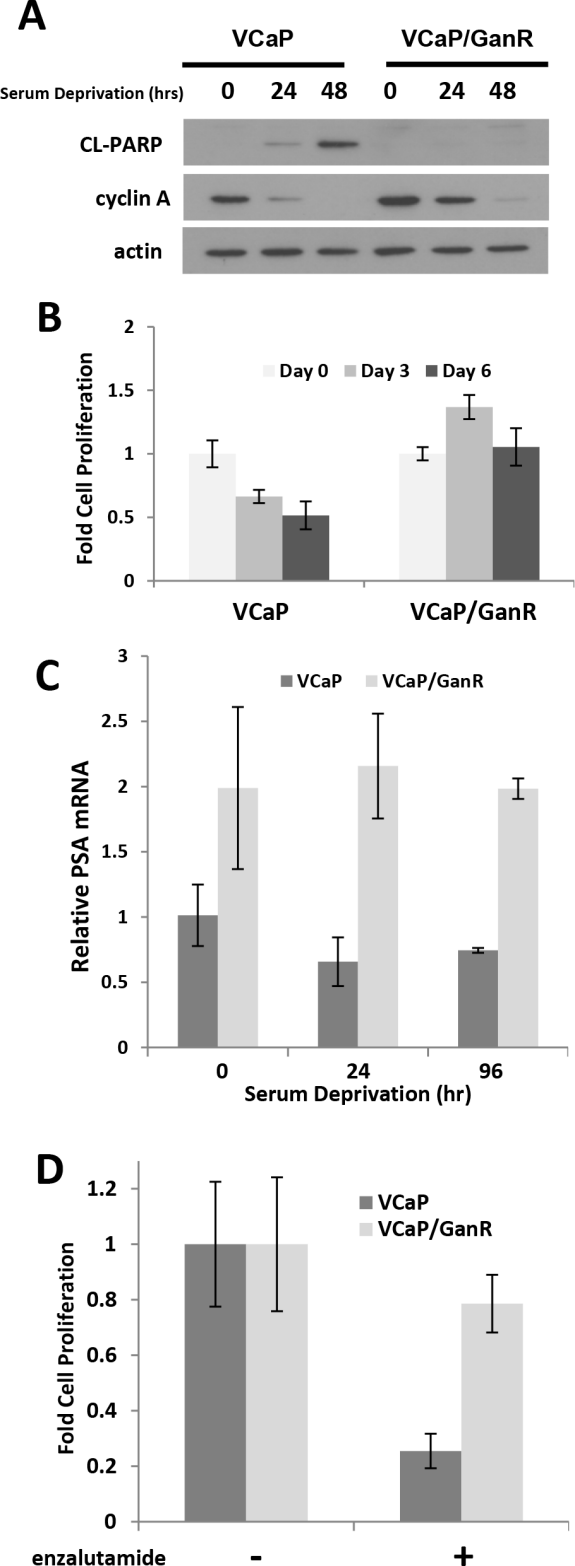
Castration-resistant characteristics of VCaP/GanR **(A)** VCaP and VCaP/GanR were cultured under androgen- and mitogen-depleted conditions in medium containing 10% CSS for the indicated times, and probed for cleaved PARP, cyclin A, and actin. **(B)** VCaP and VCaP/GanR were cultured in medium supplemented with 10% CSS and cell proliferation is shown relative to cell number at initiation of serum deprivation (Day 0). **(C)** VCaP and VCaP/GanR were grown in medium containing 10% CSS for the times indicated. PSA mRNA was assessed by reverse transcriptase realtime PCR, normalized to HPRT, and shown relative to parental VCaP. **(D)** VCaP and VCaP/GanR were treated for five days with androgen receptor inhibitor enzalutamide (500 nmol/L) in medium containing 2% FBS and proliferation is shown relative to vehicle treatment. Panels (B-D) were performed in triplicate. Data are shown ± SD.

As parental VCaP are androgen-dependent, we evaluated whether acquired resistance to ganitumab impacted the androgen-dependent phenotype. VCaP/GanR and parental VCaP were cultured in androgen- and mitogen-depleted medium containing 10% charcoal stripped serum (CSS). VCaP/GanR did not undergo apoptosis as evidenced by lack of cleaved PARP and these cells also maintained higher levels of cyclin A (Figure 2a). VCaP/GanR survived, but did not proliferate, in mitogen- and steroid-depleted medium whereas VCaP decreased in cell number showing that VCaP/GanR exhibited castration-resistant characteristics (Figure 2b).

To investigate in greater detail the castration resistant phenotype of the VCaP/GanR, we examined expression of the androgen receptor target gene *PSA,* which is indicative of receptor activity. Consistent with elevated androgen receptor activity, VCaP/GanR expressed higher levels of PSA mRNA compared to parental VCaP under both normal growth conditions as well as following serum deprivation (Figure 2c). Further, we found that VCaP/GanR exhibited less growth inhibition than VCaP after treatment with the clinically relevant androgen receptor antagonist enzalutamide (21% and 75% inhibition, respectively) (Figure 2d).

### Characterization of IGF-1R related signaling pathways in VCaP/GanR

**Figure 3:**
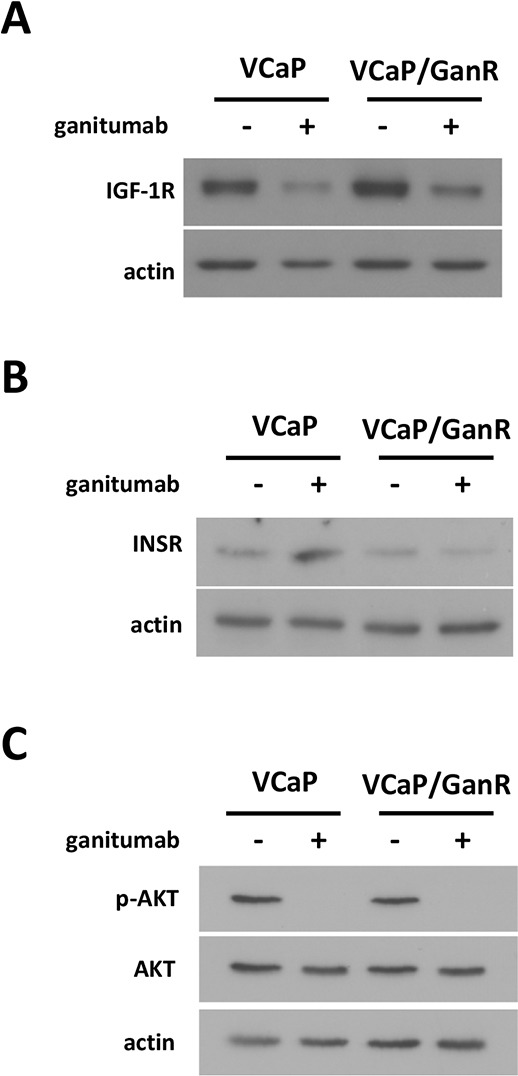
Characterization of IGF-1R-related signaling pathways in VCaP/GanR VCaP and VCaP/GanR were treated with ganitumab (500 nmol/L) or vehicle for 72 hours in medium containing 2% FBS. Lysates were immunoblotted for IGF-1R and actin **(A)**, INSR and actin **(B)**, or phospho-AKT then probed for total AKT and actin **(C)**. Panels (A,B,C) are representative of three independent experiments.

VCaP and VCaP/GanR harbored similar basal levels of IGF-1R and treatment with ganitumab reduced IGF-1R protein levels in both VCaP and VCaP/GanR (Figure 3a). The closely related RTK insulin receptor (INSR), whose overexpression is a proposed mechanism of resistance to ganitumab in Ewing's Sarcoma, was also assessed [5]. VCaP and VCaP/GanR contained similar levels of INSR. VCaP showed an acute increase in INSR after ganitumab treatment, which was not observed in the VCaP/GanR (Figure 3b). These results suggest that overexpression of either IGF-1R or INSR to compensate for loss of IGF-1R signaling after ganitumab treatment does not underlie ganitumab resistance in VCaP/GanR. Further, unlike parental VCaP, VCaP/GanR did not upregulate INSR in response to ganitumab-mediated loss of IGF-1R signaling, which may indicate acquisition of alternative growth and survival signaling.

AKT and ERK are downstream effectors of IGF-1R [6, 9] and phosphorylation status of these signaling molecules was assessed. AKT phosphorylation levels were decreased after ganitumab treatment in both cell lines (Figure 3c). ERK1/2 phosphorylation levels were similar between VCaP and VCaP/GanR and were not affected by ganitumab treatment (data not shown). These data suggest that resistance to ganitumab is not due to reactivation of downstream IGF-1R signaling pathways through alternative means.

### Phospho-proteome kinase arrays

**Figure 4:**
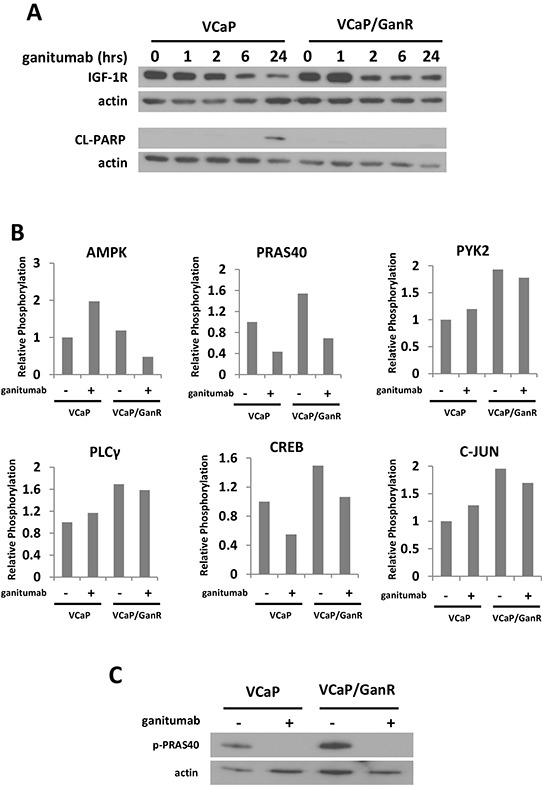
Phospho-proteome profiling of VCaP and VCaP/GanR **(A)** VCaP and VCaP/GanR were treated with ganitumab (500 nmol/L) for 0–24 hours in medium containing 10% FBS. Lysates were immunoblotted for IGF-1R, cleaved PARP, and actin. **(B)** VCaP and VCaP/GanR were treated with ganitumab (+) (500 nmol/L) or control antibody (−) for six hours followed by a phospho-proteome array with results shown relative to VCaP control antibody treatment. **(C)** VCaP and VCaP/GanR were treated with ganitumab (500 nmol/L) or vehicle for six hours in 10% FBS and lysates were immunoblotted for phospho-PRAS40 and actin.

To simultaneously assess protein phosphorylation changes that might underlie acquired resistance to ganitumab in VCaP/GanR, protein kinase phosphorylation arrays were performed. These arrays permit evaluation of the relative phosphorylation of 42 distinct signaling molecules. To determine the optimal time to examine differences in protein phosphorylation between VCaP and VCaP/GanR, a time course of ganitumab treatment (0–24 hours) was performed (Figure 4a). The arrays were conducted following a six hour ganitumab treatment as IGF-1R levels were reduced, but apoptosis had not yet increased in the control VCaP. This time frame should permit us to eliminate protein phosphorylation changes that were a consequence of apoptosis. We found changes in phosphorylation of proteins (AMPK and PRAS40) that regulate mammalian target of rapamycin (mTOR), as well as several proteins that are subject to calcium regulation or are mediators of calcium signaling including: CREB, PLCγ, PYK2, and c-Jun. Alterations in protein phosphorylation were plotted relative to vehicle treatment of VCaP (Figure 4b). Phospho-PRAS40 was also verified by immunoblot (Figure 4c).

**Figure 5:**
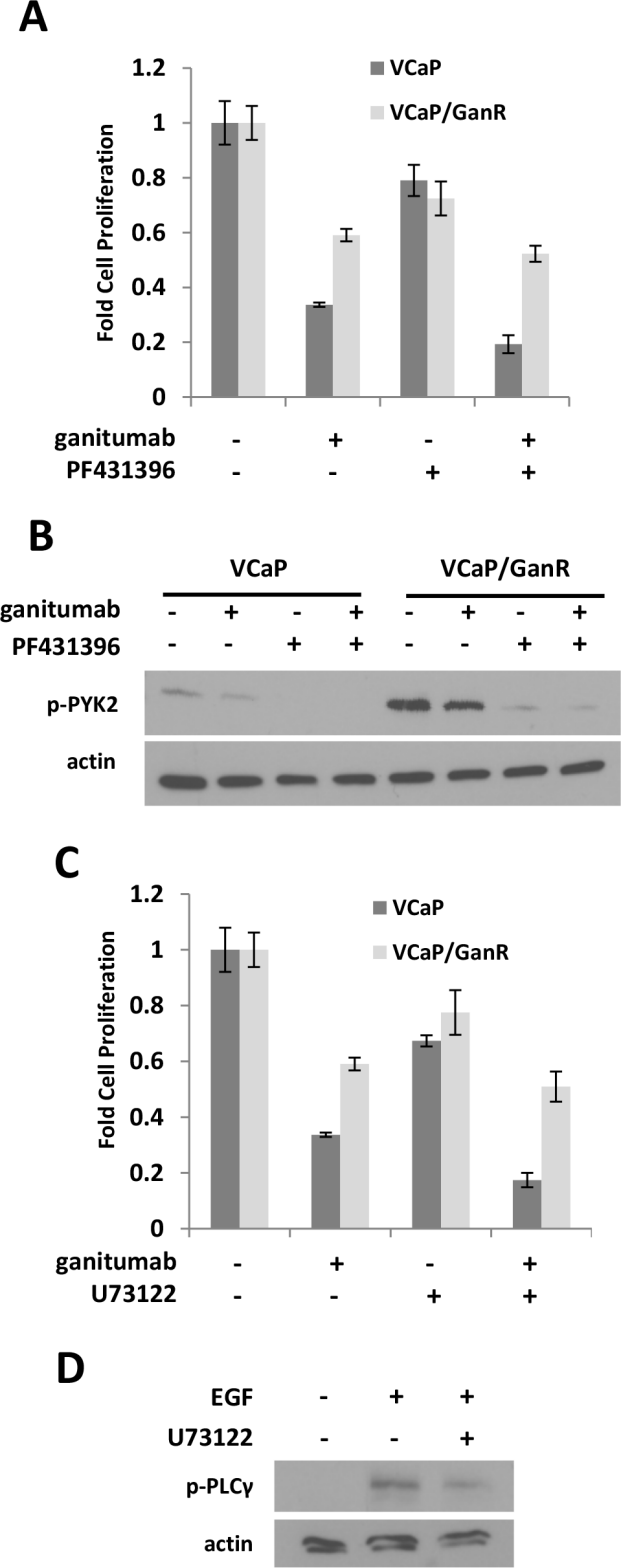
Effects of PYK2 and PLCγ inhibition on VCaP/GanR **(A)** VCaP and VCaP/GanR were treated for six days with PYK2 inhibitor PF431396 (1 μmol/L) alone and in combination with ganitumab (500 nmol/L) in medium containing 2% FBS and proliferation is shown relative to vehicle treatment. **(B)** VCaP and VCaP/GanR were treated for 1 hour with PF431396 alone and in combination with ganitumab at the above concentrations. Lysates were immunoblotted for phospho-PYK2 and actin. **(C)** VCaP and VCaP/GanR were treated for six days with PLCγ inhibitor U73122 (500 nmol/L) alone and in combination with ganitumab (500 nmol/L) in medium containing 2% FBS and proliferation relative to vehicle treatment is shown. **(D)** VCaP were treated with U73122 or vehicle for one hour, then treated with EGF (10 ng/ml) for five minutes. Lysates were immunoblotted for phospho-PLCγ and actin. Panels (A,C) are representative experiments performed in triplicate displayed ± SD.

Phosphorylation of PYK2, a critical mediator of cell migration, proliferation and survival [31] was elevated in VCaP/GanR relative to parental VCaP (Figure 4b). To assess the possible contribution of increased PYK2 signaling to ganitumab resistance, VCaP and VCaP/GanR were treated with ganitumab, the PYK2 inhibitor PF431396 alone, or in combination with ganitumab. Inhibition of PYK2 was not sufficient to restore sensitivity to ganitumab in VCaP/GanR (Figure 5a). PF431396 inhibition of PYK2 phosphorylation was verified by immunoblot (Figure 5b).

PLCγ phosphorylation was increased in VCaP/GanR compared to parental VCaP (Figure 4b). However, inhibition of PLCγ using U73122 was also not sufficient to restore sensitivity to the anti-proliferative effects of ganitumab in VCaP/GanR (Figure 5c). The U73122 concentration was sufficient to decrease EGF-induced phosphorylation of PLCγ (Figure 5d). U73122 administration alone did not induce apoptosis in either VCaP or VCaP/GanR at the concentrations tested (data not shown).

### Elevated mTOR activity does not promote acquired resistance to ganitumab

**Figure 6:**
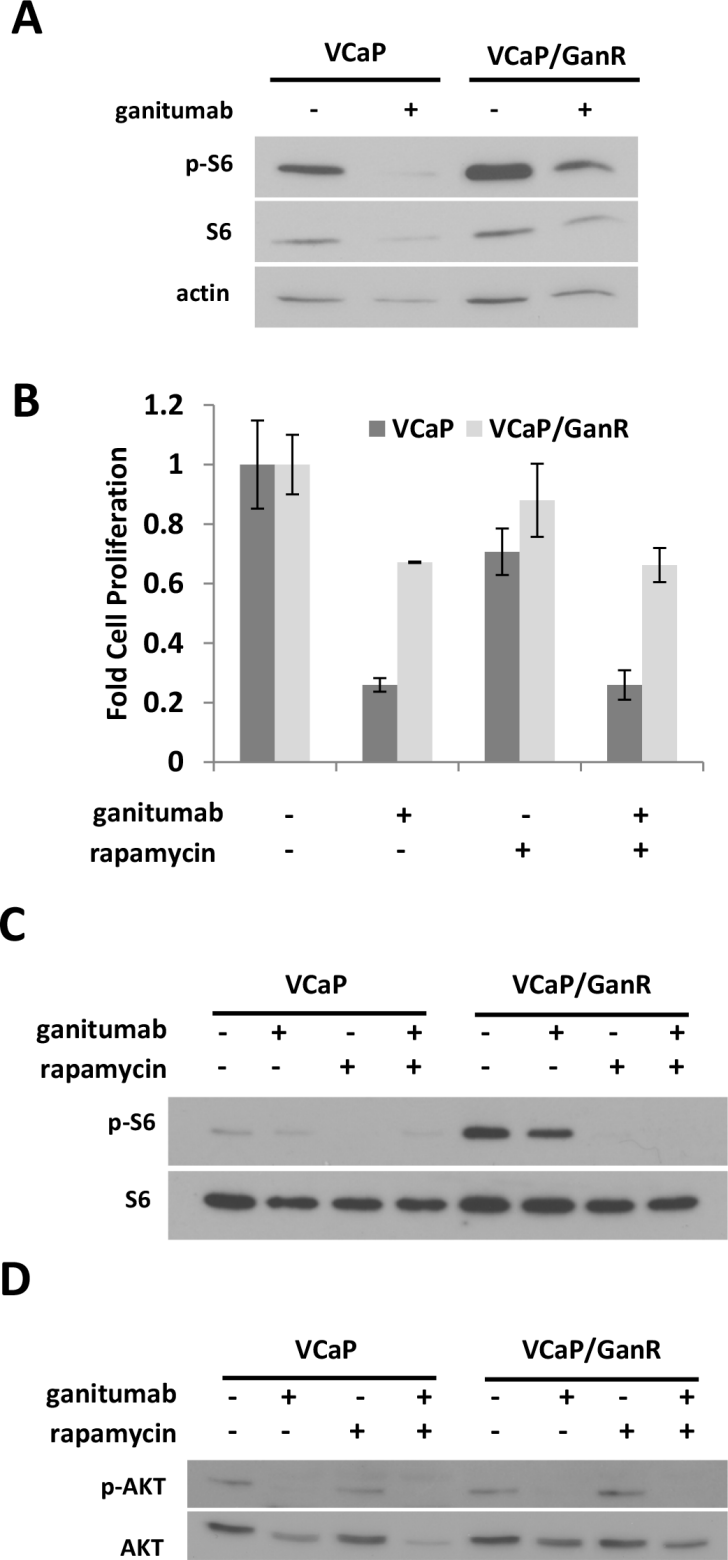
Increased mTOR activity does not contribute to ganitumab resistance **(A)** VCaP and VCaP/GanR were treated with rapamycin (50 nmol/L) alone or in combination with ganitumab (500 nmol/L) for 72 hours in medium supplemented with 2% FBS. Lysates were probed for phospho-S6 and then re-probed for total S6 and actin. **(B)** VCaP and VCaP/GanR were treated with rapamycin (50 nmol/L), ganitumab (500 nmol/L) or the combination for six days in medium containing 2% FBS and cell proliferation is shown ± SD. VCaP and VCaP/GanR were treated with rapamycin (50 nmol/L), ganitumab (500 nmol/L) or the combination for 72 hours and probed for **(C)** phospho-S6 and actin or **(D)** phospho-AKT then AKT. Panel **(B)** is representative of three independent experiments performed in triplicate. Panels (A,C,D) are representative of two independent experiments.

mTOR activity is implicated as a mechanism of resistance to growth factor receptor inhibition in several cancers. The phospho-proteomic assays showed alterations in two regulators of mTOR (AMPK and PRAS40) that have the potential to increase mTOR activity in VCaP/GanR. To assess mTOR activity, we treated VCaP and VCaP/GanR with ganitumab and monitored phosphorylated S6. S6 was highly phosphorylated in VCaP/GanR relative to VCaP following vehicle or ganitumab treatment (Figure 6a). Ganitumab treatment decreased levels of phosphorylated S6 in both parental VCaP and VCaP/GanR. VCaP/GanR-2 and VCaP/GanWD also exhibited increased phosphorylated S6 (Supplementary figure 1c) (data not shown). Thus, mTOR activity appeared to be elevated in models of ganitumab resistance.

To test whether increased mTOR activity was responsible for ganitumab resistance, VCaP and VCaP/GanR were treated with rapamycin, an mTOR inhibitor, alone and in combination with ganitumab. Rapamycin alone inhibited cell proliferation in VCaP but did not significantly decrease VCaP/GanR cell proliferation (Figure 6b). No increase in sensitivity to ganitumab was noted when rapamycin was combined with ganitumab. The concentration of rapamycin used was sufficient to decrease phosphorylated S6 in both VCaP and VCaP/GanR (Figure 6c). These results indicate that mTOR activity is likely not responsible for acquired resistance to ganitumab. AKT phosphorylation was not increased after rapamycin treatment (Figure 6d). Thus, activation of compensatory mechanisms that have been shown to increase phospho-AKT after rapamycin administration [32], are not likely responsible for the lack of ganitumab-sensitizing effects of rapamycin.

### Acquired resistance to ganitumab is calcium dependent

**Figure 7:**
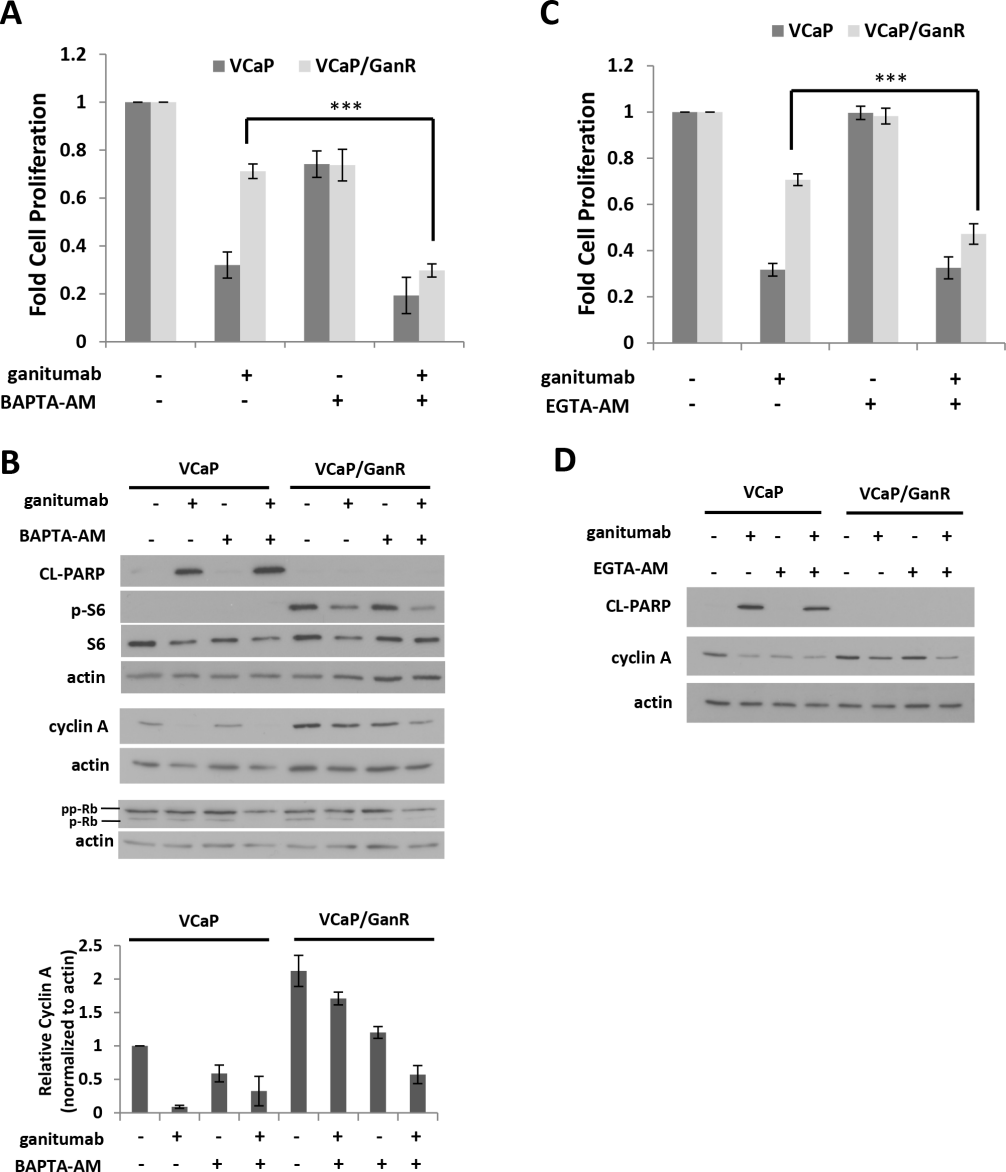
Treatment with cell permeable calcium chelators restores sensitivity to ganitumab **(A)** VCaP and VCaP/GanR were treated with BAPTA-AM (1 μmol/L) alone or combined with ganitumab (500 nmol/L) in medium containing 2% FBS for six days and relative cell proliferation is shown. VCaP and VCaP/GanR were cultured in medium supplemented with 2% FBS containing BAPTA-AM (1 μmol/L) alone or combined with ganitumab (500 nmol/L) for 72 hours. **(B)** Upper Panel: Lysates were immunoblotted for actin, cleaved PARP, and phospho-S6, then probed for total S6, cyclin A, Rb and actin. Lower Panel: Quantification of densitometry for cyclin A normalized to actin is displayed for three combined independent experiments and shown relative to VCaP vehicle treatment ± SEM. **(C)** VCaP and VCaP/GanR were treated with EGTA-AM (1 μmol/L) alone or combined with ganitumab (500 nmol/L) for six days in medium containing 2% FBS and relative proliferation is shown. VCaP and VCaP/GanR were treated with EGTA-AM (1 μmol/L) alone or combined with ganitumab (500 nmol/L) in medium supplemented with 2% FBS for 72 hours. Lysates were immunoblotted for cleaved PARP, cyclin A and actin **(D)**. Panels (A,C) represent 3 combined experiments performed in triplicate (± SD). Panels (B,D) are representative of 3 independent experiments. (***<.001, Two-tailed Student's t-test)

The phospho-proteome arrays implicated several signaling molecules that are regulated by intracellular calcium including: CREB, PLCγ, PYK2, and c-Jun. These proteins showed increased phosphorylation (Figure 4b) in VCaP/GanR relative to VCaP under vehicle and ganitumab treatments. We used BAPTA-AM, a cell-permeable calcium chelator, which decreases intracellular calcium, to evaluate the contribution of intracellular calcium levels to ganitumab resistance. While BAPTA-AM had modest anti-proliferative effects on both VCaP and VCaP/GanR as a single agent, combining ganitumab with BAPTA-AM greatly decreased cell proliferation of VCaP/GanR compared to ganitumab alone (Figure 7a). Combining BAPTA-AM and ganitumab did not have an additive inhibitory effect on cell proliferation in passage-matched parental VCaP (Figure 7a). Thus, reduction in intracellular calcium sensitized VCaP/GanR to the anti-proliferative effects of ganitumab.

We examined the effects of combined BAPTA-AM and ganitumab on apoptosis in VCaP/GanR. While ganitumab and BAPTA-AM substantially decreased cell proliferation, this drug combination did not result in increased levels of cleaved PARP in VCaP/GanR, suggesting that the decrease in cell number was not due to apoptosis (Figure 7b). In addition, phospho-S6 was unchanged in VCaP/GanR when BAPTA-AM was administered alone, and no additive effect was apparent when BAPTA-AM was combined with ganitumab (Figure 7b). These results further validate the conclusion that mTOR activity was not required for acquired resistance to ganitumab in VCaP/GanR.

Because the combination of BAPTA-AM and ganitumab inhibited cell proliferation in VCaP/GanR without affecting apoptosis, markers of G1 to S phase cell cycle progression were examined (Figure 7b). Ganitumab treatment decreased cyclin A levels in both VCaP and VCaP/GanR. However, a larger decrease in cyclin A occurred in VCaP/GanR when ganitumab was combined with BAPTA-AM. Ganitumab or BAPTA-AM alone had no appreciable effect on levels of hyperphosphorylated Rb (ppRb) in either VCaP or VCaP/GanR. However, combining ganitumab and BAPTA-AM decreased pp-Rb in VCaP/GanR, again indicative of decreased G1 to S phase cell cycle transition. These data suggest that BAPTA-AM restores sensitivity of VCaP/GanR to ganitumab through decreased S-phase entry, but not through apoptotic mechanisms.

Administration of another cell permeable calcium chelator, EGTA-AM also increased the anti-proliferative effects of ganitumab in VCaP/GanR (Figure 7c). Similar to BAPTA-AM, the combination of EGTA-AM and ganitumab had no effect on cleaved PARP but amplified the ganitumab-mediated decrease in cyclin A (Figure 7d). Lowering intracellular calcium levels by culturing cells in calcium-free medium also restored sensitivity to ganitumab in VCaP/GanR (Supplementary Figure 2). Together, these results indicate that acquired resistance to ganitumab of VCaP/GanR is dependent on intracellular calcium levels.

## DISCUSSION

While inhibition of IGF-1R using ganitumab has shown preclinical efficacy in prostate cancer, resistance occurs in some cases [13]. Understanding the mechanisms of resistance is crucial for optimizing use of ganitumab in clinical trials. Here we show the development of a stable and reproducible *in vitro* model of acquired resistance to ganitumab termed VCaP/GanR that may arise in ganitumab-treated patients. While parental VCaP underwent apoptosis in response to ganitumab, VCaP/GanR did not. Of the many pathways examined, ganitumab sensitivity was restored to VCaP/GanR only by decreasing intracellular calcium concentrations, which resulted in reduced cyclin A and hyperphosphorylated Rb indicative of impaired G1 to S phase cell cycle progression. Our results indicate that calcium-dependent pathways affecting cell cycle progression and calcium-independent avoidance of apoptosis underlie acquired resistance to ganitumab in this model.

Resistance to IGF-1R inhibition in other cancers has been attributed to increased INSR signaling [5]; however, we did not observe alterations consistent with such a mechanism. IGF-1R and INSR levels were unchanged between VCaP/GanR and parental VCaP. Phosphorylation status of major downstream effectors AKT and ERK also remained relatively unchanged between ganitumab-treated VCaP and VCaP/GanR. Long term inhibition of RTKs, such as IGF-1R, often results in compensatory signaling by other RTKs thereby restoring activity to downstream effectors such as AKT and ERK. Our data indicate that acquired resistance to ganitumab was not through compensatory RTK signaling and reactivation of downstream effectors.

Our model of acquired resistance to ganitumab showed several characteristics of castration-resistance. VCaP/GanR survived androgen-deprivation. Additionally, VCaP/GanR, compared to parental VCaP, displayed elevated androgen receptor signaling even in the absence of androgen treatment. VCaP/GanR were significantly less inhibited by androgen receptor antagonist enzalutamide than parental VCaP. Thus, VCaP/GanR exhibited castration-resistant characteristics that may have a clinical impact after ganitumab therapy.

Phospho-proteomic arrays established that two regulators of mTOR, namely AMPK and PRAS40, were altered in a manner consistent with increased mTOR activity in VCaP/GanR compared to parental VCaP. Both IGF-1R and mTOR are key regulators of cellular metabolism, thus an increase in mTOR signaling may be a compensatory response to loss of IGF-1R signaling. Increased mTOR activity was suggested as a mechanism of resistance to IGF-1R inhibition in Ewing's Sarcoma [5] as well as in a case of acquired resistance to HER2 receptor inhibitor lapatinib in breast cancer [33]. Surprisingly, elevated mTOR signaling in VCaP/GanR was not responsible for ganitumab resistance as rapamycin did not restore ganitumab sensitivity to VCaP/GanR.

We also observed increased phosphorylation of several calcium-regulated molecules in VCaP/GanR compared to VCaP including: CREB, PLCγ, PYK2, and c-Jun. Neither inhibition of PYK2 nor PLCγ restored sensitivity to ganitumab in VCaP/GanR. In contrast, when total intracellular calcium levels were decreased using a cell-permeable calcium chelator, VCaP/GanR regained sensitivity to ganitumab through decreased G1 to S phase cell cycle progression, but apoptosis was unaffected. Acquired resistance to ganitumab may be due to multiple pathways that share a reliance on intracellular calcium.

Calcium is a major regulator of cell cycle progression [34]. In non-cancerous cells, ordered control of intracellular calcium levels is crucial for regulated cell cycle progession [35]. IGF-1 signaling increases cyclin D1 and E expression, and induces calcium transients, which promote G1-S phase cell cycle progression [36–38]. Increased expression of the TRPV6 calcium channel, which would increase intracellular calcium, is associated with prostate cancer tumor progression [39]. We noted calcium-dependent cell cycle progression in VCaP/GanR that was not seen in the parental VCaP. Therefore, it is plausible that altered calcium-dependent cell cycle progression compensates for the loss of IGF-1-mediated cell cycle progression thus promoting acquired resistance to ganitumab.

Calcium regulates many processes beyond the cell cycle including proliferation, apoptosis, cellular metabolism (including the ‘Warburg Effect’), migration, and epithelial-mesenchymal transition in cancer [40, 41]. Therefore, calcium signaling in prostate cancer may represent potential new therapeutic targets. Fluxes in intracellular calcium concentrations are the result of alterations in calcium channel expression in several cancers including prostate [42–45]. Calcium channels are considered druggable targets with several small molecule inhibitors available [40]. Zhang and colleagues showed that disruption of calcium homeostasis through inhibition of the permeable ion channel TRPM8 reduced viability of LNCaP prostate cancer [45]. Reduction of intracellular calcium levels through inhibition of calcium channels or depletion of calcium stores represent treatment regimens that may be beneficial when combined with ganitumab against prostate cancer.

Here we show that decreasing intracellular calcium restored ganitumab sensitivity in VCaP/GanR prostate cancer cells. Rescue of ganitumab anti-proliferative actions was due to calcium-mediated effects on G1 to S phase cell cycle transition; however, VCaP/GanR also exhibited resistance to apoptosis that was not calcium-dependent. Combined, these alterations resulted in acquired resistance to ganitumab. IGF-1R inhibition shows efficacy against prostate cancer, but like most cancer therapies, the effectiveness of this approach will require identification of molecular features that make tumor cells susceptible. Understanding further the mechanism of ganitumab resistance is necessary for ganitumab to be more useful in the clinical setting, so that additional clinical trials will include the most sensitive patient population.

## MATERIALS & METHODS

### Cell Culture

VCaP (provided by Dr. Kenneth Pienta (Johns Hopkins, Baltimore MD) were maintained as previously described [46]. VCaP were passaged in 500 nmol/L ganitumab for 12 weeks and were termed VCaP/GanR (ganitumab-resistant). A second ganitumab resistant derivative termed VCaP/GanR-2 was derived in the same manner. Both VCaP/GanR and VCaP/GanR-2 were maintained in the presence of 500 nmol/L ganitumab. VCaP/GanR were withdrawn from ganitumab for 8 weeks and this derivative was termed VCaP/GanWD. All parental VCaP used in these studies were passage-matched with VCaP/GanR. PF431396 was obtained from Sigma Aldrich. U73122 and rapamycin were obtained from Cayman Chemicals. BAPTA-AM and EGTA-AM were obtained from Calbiochem. Enzalutamide was obtained from MedChem Express. Ganitumab and control antibody were obtained from Amgen Inc.

### In vitro proliferation assays

VCaP, VCaP/GanR, VCaP/GanR-2, or VCaP/GanWD were seeded in 24-well plates (BD Falcon) (4.5×10^4^ cells) in DMEM-H (Gibco) supplemented with 10% fetal bovine serum (FBS) (Atlanta Biologicals). The following day medium was exchanged for medium supplemented with 2% FBS and drug(s) or vehicle as indicated. Cells were incubated for six days, with media exchanged every three days. Cells were trypsinized (Cellgro), mixed with trypan blue (Gibco), and live cells were counted using a hemocytometer.

### Serum starvation cell proliferation assay

VCaP and VCaP/GanR were seeded in 3 separate 24-well plates (BD Falcon) (5×10^4^ cells) in DMEM-H (Gibco) supplemented with 10% FBS (Atlanta Biologicals). The following day cells were washed with PBS, and one plate trypsinized and counted as detailed above to serve as Day 0 timepoint. Cells were incubated with medium containing 10% charcoal-stripped serum (CSS), and a plate was counted on Day 3 and Day 6 as detailed above.

### Reverse Transcriptase quantitative PCR

VCaP and VCaP/GanR were seeded in 24-well plates (5×10^4^ cells) in DMEM-H supplemented with 10% FBS. Cells were washed with PBS and medium replaced with DMEM-H supplemented with 10% charcoal-stripped serum (CSS) for the indicated times. Cells were harvested using Trizol reagent (Invitrogen) according to the manufacturer's protocol. Total RNA was reverse transcribed using a cDNA archive kit (Applied Biosystems). Taqman probes for *HPRT* and *PSA* were purchased from Applied Biosystems. Realtime PCR was performed as previously described [47].

### Western Blots

VCaP, VCaP/GanR, VCaP/GanR-2, or VCaP/GanWD were plated at 6.5×10^5^ in the appropriate medium containing 10% FBS in 60mm plates. The following day, cell monolayers were washed with PBS, and medium supplemented with 2–10% FBS as indicated was added with treatment or vehicle. Plates were incubated for time periods as indicated, harvested in RIPA buffer and immunoblotted. Western blots were performed as previously described [47, 48]. Antibodies against phospho-AKT, total AKT, cleaved PARP, IGF-1R, phospho-S6, phospho-PRAS40, phospho-PYK2, phospho-PLCγ, and total S6 were obtained from Cell Signaling Technologies. The antibody against Rb was obtained from Oncogene Research Products. Antibodies against actin, INSR, cyclin A, and all secondary HPRT conjugated antibodies were obtained from Santa Cruz. Immunoblots were developed using an enhanced chemiluminescence detection spray (Denville Scientific). Densitometry was performed using Adobe Photoshop CS3.

### Phospho-proteome Kinase Array

VCaP or VCaP/GanR were plated at 5×10^6^ in 100 mm plates (BD Falcon) in media containing 10% FBS. The following day medium was replaced with DMEM containing 10% FBS and 500 nmol/L ganitumab or control IgG1 antibody and incubated for six hours prior to harvest. Proteome Profiler Human Phospho-Kinase Arrays ARY003B were obtained from R&D systems and performed following the manufacturer's protocol. Densitometry was performed using Adobe Photoshop CS3.

## SUPPLEMENTARY FIGURES


