# Supplementary material for: A novel calcium-dependent mechanism of acquired resistance to IGF-1 receptor inhibition in prostate cancer cells
Source: Oncotarget. 2014 Aug 19;5(19):9007–21. doi: 10.18632/oncotarget.2346 (PMC4253414; doi:10.18632/oncotarget.2346)
Supplement: Supplementary file 1 [file oncotarget-05-9007-s001.pdf]

SUPPLEMENTARY FIGURES

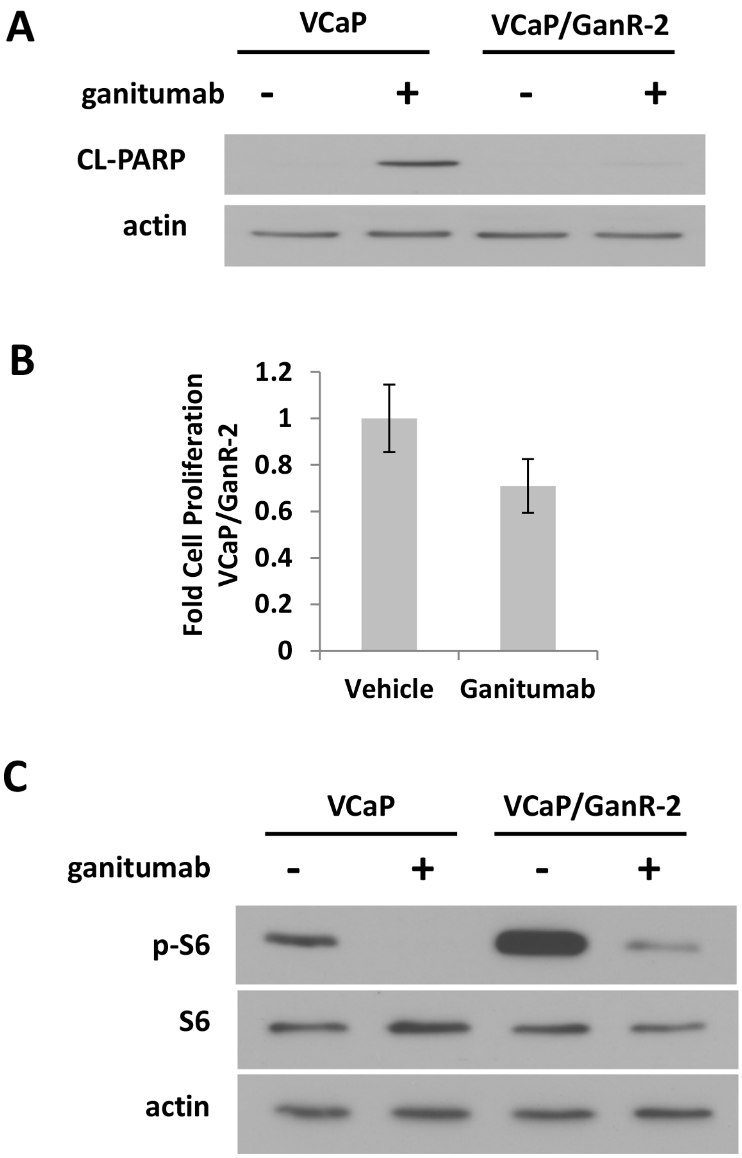

Supplementary Figure 1: (A) VCaP and VCaP/GanR-2 were treated for 72 hours in medium containing 2% FBS with ganitumab (500 nmol/L) or vehicle. Lysates were immunoblotted for cleaved PARP and actin. **(B)** VCaP and VCaP/GanR-2 were treated with ganitumab (500 nmol/L) or vehicle (PBS) for six days in medium containing 2% FBS and relative proliferation is shown ± SD. **(C)** VCaP and VCaP/GanR-2 cells were treated with ganitumab (500 nmol/L) or vehicle (PBS) in medium containing 2% FBS for 72 hours. Lysates were immunoblotted for phosphorylated S6 and actin, then stripped and probed for total S6. Panels (A,C) are representative of two independent experiments. Panel (B) is representative of two independent experiments performed in triplicate.

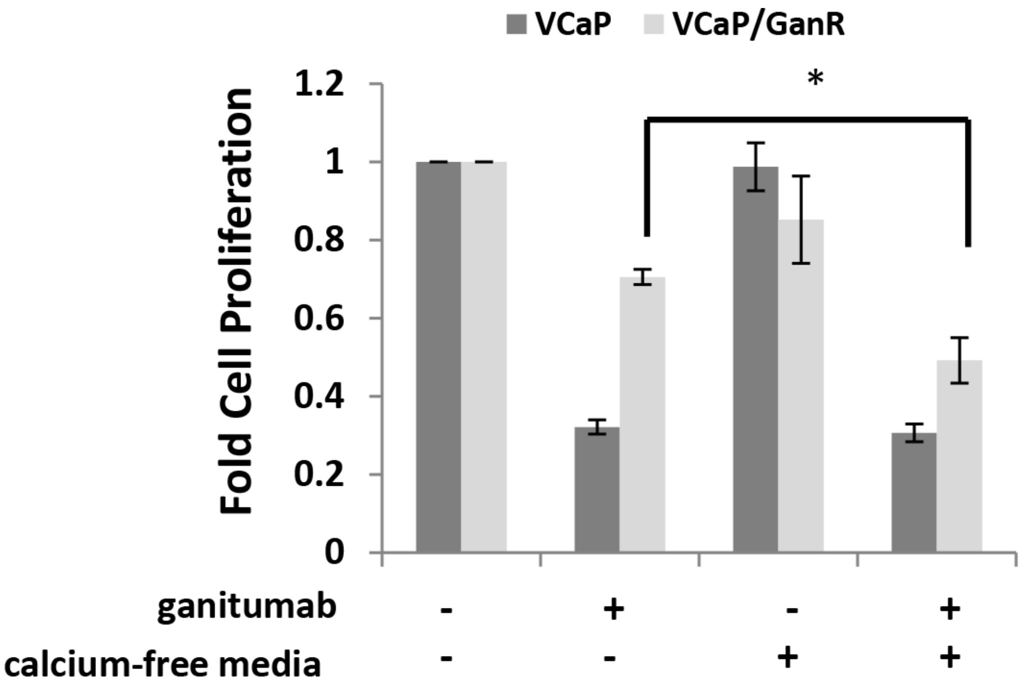

Supplementary Figure 2: VCaP and VCaP/GanR cells were treated for 72 hours with ganitumab (500 nmol/L) or vehicle in either normal medium or calcium free medium containing 2% FBS for 72 hours. Data are shown relative to vehicle treatment in normal medium ± SEM. Data represent 4 combined experiments performed in triplicate. (*<.05, Two-tailed Student's t-test)
